# Management of Pain in Children with Burns

**DOI:** 10.1155/2010/825657

**Published:** 2010-09-16

**Authors:** M. Gandhi, C. Thomson, D. Lord, S. Enoch

**Affiliations:** ^1^Central Manchester and Manchester University Children's Hospitals NHS Trust, Manchester, UK; ^2^University Hospitals of South and Central Manchester, Manchester, UK; ^3^Royal Manchester Children's Hospital, Oxford Road, Manchester M13 9WL, UK

## Abstract

Burn injuries are common in children under 10 years of age. Thermal injury is the most common mechanism of injury and scalds account for >60% of such injuries. All children with burns will experience pain, regardless of the cause, size, or burn depth. Undertreated pain can result in noncompliance with treatment and, consequently, prolonged healing. It is acknowledged that the monitoring and reporting of pain in children with burns has generally been poor. Due to the adverse physiological and emotional effects secondary to pain, adequate pain control is an integral and requisite component in the management of children with burns. A multidisciplinary approach is frequently necessary to achieve a robust pain relief. Key to successful treatment is the continuous and accurate assessment of pain and the response to therapy. This clinical review article discusses the essential aspects of the pathophysiology of burns in children provides an overview of pain assessment, the salient principles in managing pain, and the essential pharmacodynamics of commonly used drugs in children with burn injuries. Both pharmacological and nonpharmacological treatment options are discussed, although a detailed review of the latter is beyond the scope and remit of this article.

## 1. Introduction

**Figure 1:**
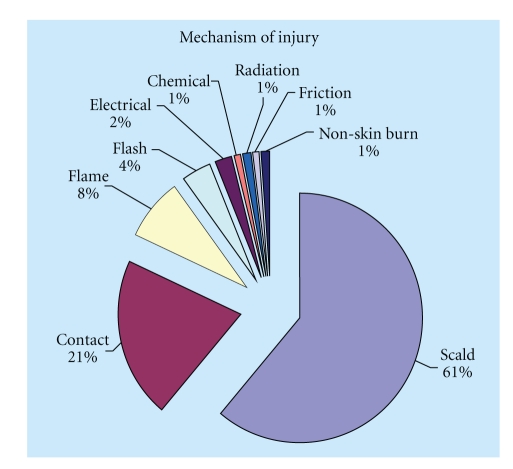
Illustration of various burn aetiologies. Note that scalds in children account for more than 60% of all burn injuries.

Children <10 years of age account for approximately 36% of burns seen in Accident and Emergency (A&E) departments [1]. About 44% of all admissions to regional burns units in the United Kingdom (UK) are sustained by those <15 years of age [2]. The aetiology of burns can be broadly divided into: thermal, electrical, and chemical injuries. Thermal injury is the most common in children with electrical and chemical injuries accounting for only 2% and 1%, respectively [2]. Thermal injuries can be further subdivided into scald, flame, contact with a hot surface, and flash burns resulting from ignition of a volatile substance. Scalds account for approximately 61% for injuries in children followed by contact burns at 21% [2] (Figure 1). 

Pain and distress are strongly associated with burns in children. Monitoring and reporting of pain in children with burns has been generally poor. For example, the potential for anticipatory pain before procedures, such as dressing changes, is high and little has been reported in the literature about chronic pain following a burn injury. Monitoring of pain is complicated by the traumatic nature of the initial injury and reaction to distress after a burn. 

Pain has adverse physiological and emotional effects, and adequate pain control is an important factor in improving outcomes. Key to successful treatment is the continuous and accurate assessment of pain, and the response to therapy. Management of pain should be a multidisciplinary approach involving a range of professionals such as the burn surgeon, paediatrician, pain specialist (usually anaesthetist), nurse, occupational therapist, physiotherapist, psychologist, play therapist, and, importantly, the child's parents/carers. This clinical review article discusses the essential aspects of the pathophysiology of paediatric burns and the effect of associated pain in the management of children with such injuries. The scientific evidence and selection criteria for the salient information and the substantiation of the information provided in this review have been obtained from sources shown in Table 1.

## 2. Pathophysiology of Burn Injury

The extent of a burn injury is determined by the degree of heat and duration of exposure of the tissue to the source [3]. The mechanism of injury can provide a useful guide to the possible severity; for example, fat scalds produce a deeper injury than water scalds due to the density. Likewise, children with other comorbidities such as paraplegia secondary to spina bifida suffer worse injury due to lack of sensation or inability to extricate themselves from the source. Local factors, such as inflammatory response and changes in perfusion also influence the final extent of the burn [3]. 

**Figure 2:**
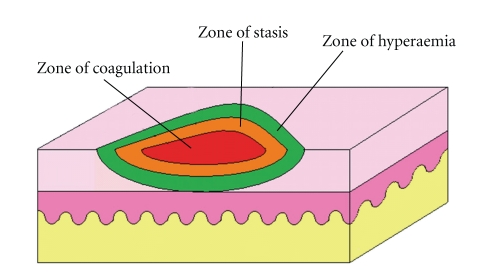
Illustration of zones of burn injury. The centre part (zone of stasis) is the worst affected and the one surrounding it (zone of stasis) is characterised by decreased tissue perfusion. The burn depth in this zone can be prevented from worsening by appropriate first aid and adequate initial fluid resuscitation.

Coagulation, stasis, and hyperaemia (Figure 2) are the three recognised zones of burn injury (Hettiaratchy and Dziewulski [4]). The zone of coagulation is where irreversible coagulation of tissue protein has occurred and this area is therefore unsalvageable. The zone of stasis is characterised by decreased tissue perfusion. Thus the aim of initial burns management is to improve blood flow to this area to prevent extension of the injury. The third zone of hyperaemia has increased perfusion and therefore is not at risk unless there are added factors such as infection [4]. 

Systemic response to a burn is associated with those affecting 30% or more of the total body surface area (TBSA) as a result of inflammatory mediator release into the circulation [3, 4]. Consequent upon this, any major system such as the cardiovascular, respiratory, renal, gastrointestinal, metabolic, or immunological can be affected. Tissue and end-organ hypoperfusion is a consequence of hypovolaemia that results from fluid loss and splanchnic and peripheral vasoconstriction. Decreased cardiac contractility and increased capillary permeability, leading to extravasation of protein and fluid into the interstitial space, also contribute to hypotension. Respiratory effects include bronchoconstriction due to inflammatory mediators and may result in respiratory distress syndrome. Basal metabolic rate (BMR) increases three-fold and there is impairment of both humoral and cell-mediated inflammatory responses [3, 4].

## 3. Burn Depths

The three main categories of burn depth are superficial, partial thickness, and full thickness. [In the US and some parts of the world, the terms 1st degree (superficial), 2nd degree (partial thickness), and 3rd degree (full thickness) are used]. Partial thickness injuries are further subdivided into superficial and deep. Currently in day-to-day clinical practice, the burn depth is assessed based on clinical evaluation using a combination of characteristics such as pain, appearance, colour, blisters (presence or absence), sensation, and capillary refill. Modalities such as Laser Doppler imaging, transcutaneous videomicroscopy (direct visualisation of dermal capillary integrity), and infrared thermography (temperature gradient between burnt and intact skin) have been attempted but are not used in routine clinical practice. Reassessment of burn depth should also be repeated 72 hours postinjury as this can change as a result of management and intervention [5]. 

Superficial burns are red and painful, only involving the epidermis, and usually heal within seven days [6]. Where there is only erythema and no epidermal loss, this is not included whilst calculating the TBSA. Superficial partial thickness injuries produce blistering and, once debrided, appear pink and wet with brisk capillary refill. These are also painful and will usually heal within 14 days. Deep partial thickness burns are less painful, have a dry and fixed blotchy red appearance, and do not blanch under pressure. Deep partial thickness burns may take longer to heal (about 21 days or more). Full thickness injuries also appear dry but with a white or brown leathery appearance. They are not painful and generally require excision and skin grafting to allow healing [3, 5]. Some of the salient features in burns of different depths are shown in Table 2.

The burn depth may also directly relate to the extent and severity of pain. The initial insult to the skin damages or destroys nerve endings but this initial stimulation causes pain regardless of the depth of burn [7, 8]. In superficial and superficial partial thickness burns, nerve endings remain intact and exposed and therefore stimulation of these, for example, from movement or touch, causes pain. In deep partial thickness injuries, some nerves may be completely destroyed and therefore the pain experienced may be less. However, it needs to be appreciated that surrounding areas (zone of stasis and hyperaemia) of a deep burn can be painful. Exposure of damaged nerve endings to inflammatory mediators, such as bradykinin and histamine, leads to hypersensitivity so that normally nonpainful stimuli cause pain [9]. In addition, the treatment/therapy instituted to treat burns such as debridement, dressing changes, and physiotherapy, leads to continued stimulation of nociceptors and, consequently, pain.

In areas of full thickness burns, all nerve endings have been destroyed and therefore this area should be insensate. Similar to deep partial thickness burns, the surrounding damaged tissue may have intact but damaged nerve endings that are still sensitive to both inflammatory and external stimuli [7–9]. Children with more severe burns are also subjected to more dressing changes, both in frequency and duration, and are also more likely to require operative management. Full thickness injuries often require grafting and donor sites may actually be more painful than the initial burn [7, 8].

## 4. General Management of Burn

The appropriate first-aid in all forms of thermal burns is to run the burnt area under a cold tap for 20 minutes. Care has to be taken in children to avoid hypothermia; therefore very cold water and ice should be avoided as these can cause vasoconstriction, making the depth of injury worse [10]. In minor burns, the injury is cleaned and blisters debrided to allow full assessment of the wound after appropriate analgesia and sedation if needed. Choice of dressing for superficial partial thickness wounds includes simple nonadherent dressings that can be used in conjunction with antimicrobial agents [6] (e.g., a nonadherent silicone dressing such as Mepitel along with betadine ointment). Tissue-engineered skin substitutes, such as Biobrane, or entirely synthetic equivalent dressings, such as Suprathel, adhere to the wound and gradually peel off as reepithelialisation occurs underneath. The main advantage of these dressings is that it can be left intact until the wound heals (provided there is no underlying infection) and only the outer dressings changed, thus reducing pain [11]. 

Deep dermal burns initially require daily dressing changes (due to increased exudation from the wound) and as healing progresses the frequency of dressing can be gradually reduced. Full thickness burns re-epithelialise only from the edges due to a lack of skin appendages that harbour epithelial cells. This makes healing very slow and therefore almost all full thickness burns, apart from very small areas (less than about 1%-2%), require excision and grafting. To minimise scarring, the aim should be to re-epithelialise the area within about 21 days of injury and therefore early excision and grafting is strongly recommended [6].

In major burns, management should follow trauma resuscitation guidelines including assessment of potential airway problems, particularly in children with facial or flame burns. Children with a burn >10% of the TBSA require fluid resuscitation, with the most commonly used formula worldwide being the Parkland formula [12]. This is calculated as 4 mL/kg/%TBSA total of Hartmann's solution over 24 hours with half given in the first 8 hours and half in the subsequent 16 hours. This formula should not be considered moralistic but rather as a guide that should be used in conjunction with the patient's physiological parameters and the volume of fluid instituted should be tailored accordingly.

Nutritional support is also vital as the BMR can increase by up to 40% after a significant burn. This catabolic state may last for as long as two years, which is of particular concern in the children as this may affect growth [13]. Although it is generally accepted that nutritional support should be started early postinjury [14], with the enteral route being preferable [15], a recent Cochrane review did not find evidence for, or against, either of these in children [16].

## 5. Management of Pain

All children with burns will experience pain, regardless of the cause, size, or depth of the burn. Undertreated pain can result in noncompliance with treatment and, consequently, prolonged healing. This can disrupt care and increase the risk of posttraumatic stress disorders. It is possible to ensure better pain management by trying to understand the child's experience rather than just acknowledging the pain. Thus the most fruitful approach would seem to be frequent assessment of pain with readiness to try alternative or additional measure when relief seems inadequate. The general attitude to pain management should be presumptive and preemptive.

Multidisciplinary assessment helps to integrate pharmacological and psychological pain relieving interventions to reduce physical, emotional, and family distress. Special attention should be paid to the child's environmental conditions. For instance, a parent's presence and participation in the procedure can be highly beneficial.

Children with burns have background pain and procedural pain and it is important to differentiate between the two. Background pain, once assessed and evaluated, can be managed pharmacologically with regular analgesia whilst procedural pain requires more intense analgesia. Procedural pain is difficult to assess and is therefore frequently under treated. Poor management of pain can lead to anticipatory anxiety before future procedures and a lower pain tolerance threshold.

Chronic pain has multiple and often unclear origins. Neuropathic pain is one cause for this and develops secondary to nerve damage, abnormalities in nerve regeneration, and reprogramming of the central nervous system [17]. It can be frustratingly unresponsive to conventional treatment modalities. Adjuvant therapies such as clonidine and anticonvulsants are effective in treatment of sympathetically mediated pain. Psychological therapies to boost coping strategies and aid relaxation should be added.

Management of pain is important during all stages of treatment including in the emergency department, during procedures such as dressing changes and after discharge when complex neuropathic pain syndromes may develop [18].

## 6. Measurement Tools

A major contributing factor to poor pain management is the difficulty children have in expressing their pain and problems that the health professionals may have in interpreting and assessing this information correctly. Hence, it is vital to assess the pain accurately to gauge the severity of pain and the effectiveness of its treatment. The pain experienced by burn children also varies greatly; therefore, analgesia needs to be tailored on an individual basis. In order to achieve this aim it is essential to measure pain in a simple and reproducible manner. Various measurement tools are available for assessment of pain in children. In our institution, we tend to use the *“FLACC tool”* and the *“Faces Ladder Scale”* as these are simple, effective, and quick to use. However, irrespective of the tool adopted, the frequency of measurement should be tailored to the appropriate stage in the burn management. During resuscitation, hourly scores must be recorded to address any breakthrough pain. This schedule can be eased whilst the background pain is monitored, but the frequency of monitoring is increased should any new event occur. Once the immediate need has been controlled with parenteral opiates, a background control primarily with paracetamol and if necessary NSAIDs can be established. The intravenous (IV) route should be used if the oral route is not appropriate (such as due to gastric intolerance or minimum fasting prior to a procedure requiring sedation). 

### 6.1. FLACC Tool (Face, Legs, Activity, Cry, and Consolability)

Whenever possible the child's self-report should be used to assess pain. However, there are situations where this may not be possible, for example, in infants or in those with cognitive impairment or language difficulties. In such instances, the FLACC tool [19] should be used (Table 3). FLACC is a behavioural assessment tool with five categories where each category scores on a scale of 0–2, which results in an overall score of between 0–10. The child should be observed for 2–5 minutes and their body activity, face, and cry noted according to the scale. If necessary, the health professional should attempt to console the child. The child's pain score should be assessed and recorded at regular intervals, especially before and after analgesia or after nonpharmacological intervention. The FLACC tool has been adequately validated in areas such as paediatric theatre recovery, and oncology and paediatric intensive care units [20].

### 6.2. Faces/Ladder Scale

**Figure 3:**
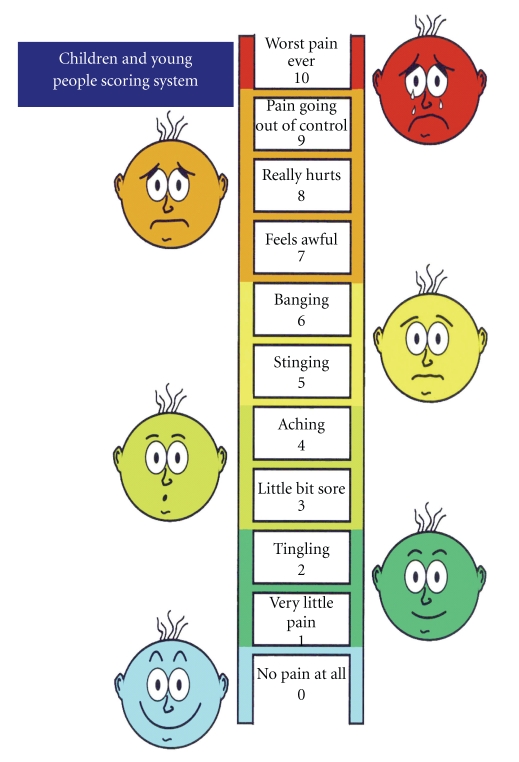
Paediatric Pain Assessment: FACES ladder. Useful in children ≥4 years of age.

Studies have found that children as young as three years old can communicate and make judgements about their pain [21]. Wong-Baker FACES Pain Rating Scale is recommended for children ≥3 years of age [22]. The faces/ladder scale should be explained to the child, that is, that the smiling faces indicates no pain whilst the distressed face indicates severe pain (Figure 3). The wording down the centre of the ladder can be read by the older child. What the child states as their pain score—either from using the numbers or faces on a scale of 0–10—should then be documented.

## 7. Pharmacological Treatments

The ideal analgesic agent in a child with burn would be the one with the following characteristics: (i) easy to administer, (ii) well tolerated, (iii) produces rapid onset of analgesia with a short duration of action, and (iv) has minimal side-effects to allow rapid resumption of activities and oral intake. Various routes such as parenteral, oral, and intranasal route are available for administration of analgesia. In the acute setting, drugs, preferably, should be given by IV route. However, intranasal route is a sound alternative. Potential advantages of intranasal route compared with parenteral or oral administration include avoidance of painful injection, avoidance of risks associated with IV access, rapid onset and titration to effect, and good bioavailability [23]. Although small trials suggest that intranasal opioids play a useful role in pain management, large clinical trials with Level 1 evidence is required to identify its advantages, safety, and acceptability.

## 8. Specific Analgesics

**Paracetamol (Acetaminophen)**

continues to be a useful first-line analgesic in minor and superficial burns. Paracetamol, a p-aminophenol derivative that exhibits analgesic and antipyretic activity [24], does not possess anti-inflammatory activity. Paracetamol acts both centrally and peripherally to produce analgesia. The IV route allows rapid passage of paracetamol in the systemic circulation leading to a rapid onset and faster distribution resulting in higher plasma concentration as compared with oral and rectal route. The IV preparation is a good adjunct along with opioids in the acute setting. Used along with opioids, it has a synergistic effect. Meyer et al. [25] described the use of paracetamol in the treatment of background pain in children (*n* = 395) after acute burn injury and found that in 50% of these children, especially the youngest and those with smaller burns, did not require any morphine.

**NSAIDs**

have analgesic and anti-inflammatory properties. Their mechanism of action is via nonselective inhibition of prostaglandin and thromboxane synthesis via inhibition of the cyclooxygenase enzyme (inhibits platelet aggregation and renal prostaglandin production). Judicious use of NSAIDs can be opioid sparing [26] but their side effects can be a limiting factor [27].

**Opioids**

provide analgesia via a variety of central and peripheral opioid receptors, particularly via the “mu” and “kappa” receptors.

**Morphine**

has the lowest lipid solubility of all the opioids, which accounts for its slow entry into the brain and subsequent delayed onset of clinical effect. Its peak analgesic effect occurs 10–20 minutes after IV administration of a bolus dose of 0.1 mg/kg. While administering morphine as continuous infusion, younger children should be managed in a High Dependency or Intensive Care area. (Dosage for children <6 months of age is 0–12.5 *μ*g/kg/hour and for children >6 months of age is 0–25 *μ*g/kg/hour.) Rate and dosage should be adjusted according to child's pain and sedation scores.

Morphine PCA can be used in children ≥5 years who have the ability to understand the workings of a PCA [28]. Bolus dose is usually 20 *μ*g/kg with a lockout interval of five minutes and background infusion of 4 to 8 *μ*g/kg/hour. In children who have difficulty pressing the “demand” button, this modality may be inappropriate. In this instance, it can be delivered by NCA (nurse controlled analgesia)—usually in a high dependency setting. Bolus dose is 20 *μ*g/kg with a background infusion of 0–20 *μ*g/kg/hour and a lock out interval of 20–60 minutes. Criteria for administration of a bolus dose are if the pain score is seven or more on a scale of 0–10 and the sedation score no greater than one. Respiratory rate should be above minimum rate for the age of the child and oxygen saturation must be monitored by continuous pulse oximetry.

**Oxycodone**

is a new semisynthetic opioid with a better bioavailability than morphine, and thus an effective alternative. Sharar et al. [29] compared oral transmucosal fentanyl citrate (OTFC, 10 *μ*g/kg) and oral Oxycodone (0.2 mg/kg) in 22 paediatric outpatient wound care procedures and concluded that OTFC and oral Oxycodone are safe and effective analgesics in the setting of monitored outpatient wound care in children.

**Fentanyl**

a synthetic, potent narcotic analgesic with potency up to 100 times that of morphine, is highly lipid soluble and has a rapid onset of action (1-2 min). The duration of analgesia is about 60 minutes. Possible side effects include hypotension, bradycardia, apnoea, chest wall spasm, muscle rigidity, and respiratory depression.

Fentanyl lozenges [30] are a solid formulation of fentanyl citrate on a stick in the form of a lollipop that dissolves slowly in the mouth for transmucosal absorption. Doses around 15–20 *μ*g/kg seem satisfactory and provide rapid onset (10 min) of pain relief [31]. In children, 10 *μ*g/kg is equianalgesic to Oxycodone 0.2 mg/kg [29].

Intranasal fentanyl has been shown to be equivalent to oral morphine in the provision of analgesia for burn wound dressing changes in children. Intranasal fentanyl may be a suitable analgesic agent for use in paediatric burns dressing changes either alone or in combination with oral morphine as a top up agent [23].

**Alfentanil**

is a short acting opioid with the peak effect reached within a minute. It undergoes hepatic metabolism to inactive metabolites that are excreted via the kidneys; it may thus be a safer option in children with impaired renal function. Change of burn dressings may require strong analgesia for a short duration of time. Studies have shown that Alfentanil can be used for this purpose as target controlled infusion [7, 8] or as a PCA [32].

**Remifentanil**

is a novel; ultrashort-acting esterase metabolised synthetic opioid. It is a selective “mu” opioid agonist and has an ester linkage rendering it susceptible to rapid metabolism by nonspecific blood and tissue esterases. Adult pharmacokinetic studies have shown a rapid onset of peak effect (blood-brain equilibration time: 1.2–1.4 min), a short duration of action independent of the duration of infusion (context sensitive half time: 3 min), and rapid clearance (40 ml kg^−1^ min^−1^). Remifentanil has been used for postoperative analgesia in neonates and has been found to have a similar pharmacological profile in neonates to that of older children and adults. Le Floch et al. [33] identified Remifentanil on its own to be a useful agent for undertaking dressing changes in spontaneously breathing, nonintubated burn patients. Due to its pharmacological profile of rapid onset and ultrashort duration of action, it is well suited for procedure related analgesia.

**Methadone**

is a synthetic opioid that provides analgesia not only as mu-opioid agonist but also acts as antagonist at the N-methyl-D-aspartate (NMDA) receptors. It has got excellent bioavailability and a prolonged duration of action. It has been found both safe and effective in management of paediatric burns [34].

**Ketamine**

acts both in the central and peripheral nervous system. It exerts strong adjuvant analgesic properties by inhibiting the binding of glutamate to the NMDA-R receptor. This mode of action is different to the action of opioid drugs such as morphine and therefore the use of ketamine in combination with morphine can improve pain relief. Ketamine in combination with morphine reduces the need for high dose of morphine to be used [35] and therefore minimises side effects [36]. Ketamine was extensively used during burn dressing changes [37] but its psychological side-effects have limited its use. All children on ketamine infusion must have a ketamine infusion observation chart and be monitored with continuous pulse oximetry.

### 8.1. Alpha 2 Adrenergic Antagonists

Maintaining appropriate sedation and analgesia in children with burns can be quite challenging and often requires high doses of analgesics and anxiolytics because tolerance develops quickly. Escalating doses of opioids and benzodiazepines provide little additional benefit while increasing the incidence of side effects. Clonidine acts by augmenting descending inhibitory spinal cord pathways. The dose used in paediatric practice is 1–3 *μ*g/kg three times a day orally or IV [38]. Clonidine is known to reduce the need for morphine in the management of postoperative pain. The addition of clonidine to the pharmacological treatment of burn pain offers a possible adjunct to the standard opioid and benzodiazepines regimen. When clonidine is no longer required the dose must be reduced gradually to avoid withdrawal and rebound hypertension. Dexmedetomidine is a novel alpha 2-adrenergic agonist that provides sedation, anxiolysis, and analgesia with much less respiratory depression than other sedatives [39].

### 8.2. Antidepressants and Anticonvulsants

These may be beneficial to improve the sleep patterns. Antidepressants appear to enhance opiate-induced analgesia while anticonvulsants are useful in the treatment of sympathetically maintained pain following burns.

**Amitriptyline**

a tricyclic antidepressant, acts by augmenting the descending inhibitory pain pathways in the spinal cord. When used in low doses, it has an established role in the management of neuropathic pain [38]. It has been shown to be effective in phantom limb pain in children [40].

**Gabapentin**

has established efficacy in the reduction of burn-induced hyperalgesia. It binds to presynaptic calcium channels involved in pain hypersensitivity and indirectly inhibits NMDA receptor overactivation [41]. Gabapentin is started at 10 mg/kg and titrated up to 40–50 mg/kg/day [38]. Recent studies have found gabapentin [42] to be useful in the management of neuropathic pain following burn injury but further research is required to define its precise usage. On a different role, Gabapentin has also been found to be effective in the management of itch in children (common after burn injury) unresponsive to simple anti-itch medications such as chlorpheniramine and trimeprazine [43].

**Entonox**

a homogenous gas made of 50% nitrous oxide and 50% oxygen, is a potent analgesic that may be used for changing the burn dressing in some conscious children [44]. It is self-administered using a demand apparatus that safeguards against inadvertent overdose. Entonox is quick acting due to the insoluble nature of nitrous oxide and wears off rapidly once administration ceases. It can either be used alone or in conjunction with other analgesics. Entonox is contraindicated in situations such as decreased consciousness, pneumothorax or air embolism (where expansion of the air trapped within the body might be dangerous), or gross abdominal distension.

### 8.3. Local Anaesthetics

These agents act by inhibiting sodium ion flux across the axonal membrane and prevent the nociceptors signalling pain reaching the central nervous system. Addition of adrenaline (1 : 200,000) produces vasoconstriction and decreases systemic absorption, thus leading to prolonged duration of action. Techniques include local infiltration and specific nerve blocks (usually performed under sedation-analgesia). IV infusions of lignocaine have been shown to be effective in alleviating neuropathic pain, especially if there is nerve damage [45]. However, a Cochrane review by Wasiak and Cleland [46] did not demonstrate any conclusive effect in patients with burns.

### 8.4. Challenges due to Pharmacokinetic and Pharmacodynamic Response to Drugs

Children with burns often show an altered pharmacokinetic and pharmacodynamic response to drugs as a result of physiological/pathological changes due to altered haemodynamics, protein binding and/or increased extracellular fluid volume, and possible changes in glomerular filtration. Hypovolaemia and depressed myocardial function leads to decreased organ and tissue perfusion, delaying absorption of oral drugs. During the hyper metabolic phase, there is increased blood flow with a rapid onset of inhaled and IV agents. Plasma albumin is decreased and this results in an increase in the free fraction of protein bound drugs. Increase in *α*1 acid glycoprotein leads to a decrease in free fraction of drugs bound to this molecule, for example, muscle relaxants. There is an increased requirement of opioids and sedatives. Tachyphylaxis and tolerance develop quickly.

## 9. Non Pharmacological Treatments

Various nonpharmacological strategies such as education (understanding of the condition), distraction, relaxation, cutaneous stimulation, acupuncture, bio-feedback, hypnosis, imagery, cognitive, and behavioural techniques can be employed to treat the pain associated with burns. A good understanding of the procedure helps children to control their anxiety cognitively, thus, helping to gain a level of pain relief. This not only corrects misconceptions and decreases anxiety but also allows children to play an active role in the procedure and to benefit fully from pain-reducing strategies. Distraction techniques such as talking, singing, praying, describing photographs, listening to music, and playing games reduces the perception of pain by stimulating the descending control system that leads to painful stimuli being transmitted to the brain [47]. Used in conjunction, these modalities help reduce analgesic requirements [48].

## 10. Conclusion

The experience of pain varies greatly between children with burn injuries. This may be related to physical factors such as size and depth of burn, as well as to the psychological and emotional support provided by the family. Accurate assessment of pain and an evaluation of the effectiveness of analgesia are vital. Various tools are available to aid in the assessment of pain in children who may have difficulty in communicating their needs. A wide variety of pharmacological interventions exist that range from simple paracetamol to sedating anaesthetic drugs. Many times, a combination of these is required to achieve robust analgesia in treating both background and procedural pain. Non-pharmacological treatments also play a role in reducing analgesic requirements. Psychological strategies should be considered to be helpful adjuncts rather than a substitute to conventional analgesics.

In summary, healthcare professionals need to acknowledge and appreciate the significance of pain associated with burn injuries in children and be aware of the various pharmacological and non-pharmacological options. Judicious use of the drugs tailored to meet the needs of individual children coupled with a multidisciplinary approach is frequently necessary to achieve optimal outcomes.

## Figures and Tables

**Table 1:** Sources of scientific evidence and selection criteria.

The scientific evidence for the preparation of this article was obtained by searching Medline, Ovid, *Burns* and the Cochrane library until June 2010 for randomised controlled trials, systematic reviews, evidence reports, and recent evidence-based guidelines from International Burn and Pain Management Associations.

**Table 2:** Some salient features of varying burn depths and their approximate healing times.

Burn depth	Appearance	Blistering	Sensation	Approximate healing time
Epidermal	Red	None	Painful	7 days
Superficial partial thickness	Pink with wet appearance. Brisk capillary refill	Blisters present	Painful	14 days
Deep partial thickness	Pale or fixed red staining. Poor capillary refill	Blisters may be present	Painful usually but can painless	21 days; may require excision and skin grafting
Full thickness	Leathery white or brown	None	None in burnt area	Usually requires excision and skin grafting

**Table 3:** Scoring system for infants, young children, cognitively impaired children, anxious children, and any child unable to use faces ladder. Paediatric Pain Assessment: FLACC scale. This pain assessment tool can be used in children <4 years and those with cognitive impairment or unable to use the “FACES” ladder.

FLACC Scale			
	0	1	2
Face	No particular expression or smile	Occasional grimace or frown, withdrawn, disinterested	Frequent to constant frown, clenched jaw, quivering chin
Legs	Normal position or relaxed	Uneasy, restless, tense	Kicking, or legs drawn up
Activity	Lying quietly normal position	Squiring, shifting back and forth, tense	Arched, rigid, or jerking
Cry	No cry (awake or sleep)	Moans and whimpers, occasional complaint	Crying steadily, screams or sobs, frequent complaints
Consolability	Content, relaxed	Reassured by occasional touching, hugging or being talked to, distractable	Difficult to console or comfort

Each of the five categories Face (F), Legs (L), Activity (A), Cry (C), Consolability (C), is scored from 0–2. This results in a total score of 0–10.
